# Predisposing and Overall Effects of Reproductive Hormones on Breast Cancer: A Review

**DOI:** 10.7759/cureus.45956

**Published:** 2023-09-25

**Authors:** Aditya K Sarda, Sangita D Jogdand

**Affiliations:** 1 Medicine and Surgery, Jawaharlal Nehru Medical College, Datta Meghe Institute of Higher Education and Research, Wardha, IND; 2 Pharmacology and Therapeutics, Jawaharlal Nehru Medical College, Datta Meghe Institute of Higher Education and Research, Wardha, IND

**Keywords:** ovarian hormones, uterine hormones, hypothalamic and pituitary hormones, placental hormones, breast cancer

## Abstract

Cancer, the second leading cause of mortality worldwide, has been the subject of extensive and quickly changing scientific study and practice. Cancer remains a mystery despite the enormous effort put into understanding the genesis of cancerous cells, the development of malignant tissues, and the process by which they propagate and recur. Cells from humans that have been recruited by cancer and, to some extent, changed into pathogenic organisms or the foundation of tumors serve as agents of destruction. Understanding cancers leads to challenging philosophical issues since they undermine and use multicellular organization processes. Cancer metastasizing cells adopt new phenotypes while discarding previous behaviors. The absence of comprehensive knowledge of this has hampered the development of therapeutics for metastatic illness. For systems-level experimental and computational metastasis modeling, integrating these complex and interconnected features continues to be a problem because metastasis has typically been studied in separate physiological compartments. Lung, breast, and prostate cancers accounted for the bulk of the 18 million new cases of cancer that were diagnosed in 2018. The most frequent cancer in women is breast cancer. Animal experimentation plays a significant role in primary and translational breast cancer research. In theory, such breast cancer models should be comparable to breast cancer in humans in terms of tumor etiology, biological behavior, pathology, and treatment response.

## Introduction and background

Epidemiologic research has dramatically benefited the current understanding of genetic and environmental carcinogenic factors for breast cancer. Breast cancer risk factors include race, familial history of the disease, ethnicity, genetics, and modifiable exposures such as increasing alcohol use, inactivity, exogenous hormone use, and unique female reproductive variables [1]. Carcinogenesis is related to changes in DNA methylation patterns. For instance, retinoic acid receptors-2 (RARb2) and adenomatous polyposis coli (APC) gene promoters were shown to be methylated in more than 90% of breast cancer patients. Hyaluronic glucosaminidase 2 (HYAL2) and hypomethylation of S100 calcium-binding protein P (S100P) were shown to be connected with breast cancer patients from the 11 to 18 years age group. Since their discovery, non-coding RNAs such as microRNAs (miRNAs) and circular RNAs (circRNAs) have demonstrated potential non-invasive diagnostic and prognostic performance of breast cancer [2]. The majority of breast cancers depend on hormones. The promoter impact of hormone therapy (including menopause and contraceptive hormone therapy) on preexisting lesions reduces risk upon treatment cessation. Hormonal contraception somewhat raises the risk among current users. Still, the risk amplitude stays low until age 40, when it becomes more noticeable due to the higher incidence of breast cancer [3]. Breast cancer development is impacted by the hormones and cytokines secreted by the placenta. We have previously shown that the human placental secretome promotes the survival and migration of estrogen receptor (ER)+ breast cancer cells (BCCL), whereas ER-negative tumors are more common in pregnant women. In the present work, we examined the impact of the placental secretome on BCCL, which is ER-negative [4].

The effects of hormone replacement therapy (HRT) on breast tissue may vary depending on the kinds of estrogen or progestogen, as well as the formulations, dosages, and durations. Conjugated equine estrogen (CEE) appears to have a lower profile risk when compared to astro-progestin treatment (HR 0.79, CI 0.65-0.97), whereas progestins with a structural affinity for testosterone are associated with a greater risk (RR 3.35, CI 1.07-10.4). The relationship with progestins, particularly in continuous combination regimens, seems to lower the risk of hyperplasia of the endometrium and cancer. In contrast, prolonged unopposed endometrial interaction with estrogen raises those risks (RR 0.71, CI 0.56-0.90) [5]. Furthermore, progesterone receptors (PR) are crucial to developing and spreading breast cancer. As a result, several progestins and antiprogestins have been created and are now being researched and evaluated in clinical studies for cancer treatment. The function of PR in carcinogenesis and breast cancer growth has been discussed in recent reviews. In this review, the emphasis has been placed on emphasizing the still unsolved issues with hormonal therapy involving PR isoform and cancer of the breast prognosis in addition to providing an overview of PR activity in normal and tumor breasts [6].

Impact of neoadjuvant chemotherapy (NACT) on the hypothalamic-pituitary axis and the resulting change in gonadotropin levels in premenopausal breast cancer patients. The outcomes for postmenopausal women having breast cancer who received NACT, namely the considerable drop in gonadotropin hormone-releasing hormone (GnRH) and follicle-stimulating hormone (FSH) concentrations comparable to control postmenopausal women, are different from those for premenopausal women [7]. Breast cancer has a complex and multifaceted etiology. Breast cancer may be caused by mutations in the BRCA1 gene, a family history of the disease, or mutagens that can harm DNA. It may also share ovarian cancer's hormonal etiology. Estrogen significantly impacts the development, differentiation, and operation of breast tissue. Adipose tissues contain the enzyme aromatase, which aids in converting circulating cholesterol to estradiol. The amount of estradiol in older women's breast tissues, particularly after menopause, is expected to be greater than the plasma circulating level due to the more significant proportion of fat cells in those tissues. The high levels of estradiol in breast tissues can have varied impacts on the ER expression in specific tissues, which can change how cancer cells behave. Because stromal cells in the breast tissues govern normal and malignant epithelial cell development by secreting growth hormones in response to endogenous hormone levels, they can also encourage metastatic activity [8].

## Review

Placenta

Human Placental Lactogen (HPL)

Placental lactogen levels may be less than controls in the early stages of pregnancy (possibly illustrating delayed placental development) and more significant compared to authorities in the later stages of pregnancy (likely consistent with higher placental masses); however, there is no direct correlation between absolute HPL concentrations and maternal glycaemic results in gestational diabetes mellitus (GDM) or pre-gestational diabetes mellitus (PGDM), nor with GDM status or risk. Additionally, in pregnancies impacted by GDM or PGDM, HPL is probably favorably associated with placental mass and child birthweight and may play a functional role in the control of fetal development. Despite being out of common clinical usage in recent years, HPL may be worth looking at again as a potential antenatal indication for diagnosing macrosomia [9]. According to studies, having a baby at full term lowers the chance of breast cancer. Studies have shown that HPL had a role in this protective effect because it was present in much higher quantities near the end of gestation [10].

Human Chorionic Gonadotropin (hCG)

Testing for hCG shows that this enzyme is involved in many functions. While its primary job is to sustain early gestation, the findings of testing may result in inaccurate inferences about the pregnancy status. This might have significant emotional and monetary repercussions for patients in various circumstances. Additionally, hCG testing might detect another disease, such as cancer [11]. Ectopically expressed chorionic gonadotropin and heterodimeric placental HCG hormone have opposite impacts on the growth of breast cancer. The causes of these conflicting effects are still up for debate. The glycosylation condition of HCG, which has been marked to increase cancer cell invasion, proliferative division, and metastasis, may be one cause. It is believed that the luteinizing hormone/human chorionic gonadotrophin receptor (LHCGR) has many polymorphisms that may also contribute to the different effects of hCG on cancerous breast cells. As a result, sensitivity and the expression of the plasma membrane might fluctuate, resulting in variable receptor activity. Additionally, there are several polymorphisms in the hCG genes, which produce various hCG subtypes [12].

Uterus

Prolactin (PRL)

Although it has been linked to several bodily processes, including metabolism and energy balance, PRL is named for its critical role in stimulating milk production during lactation. Many of the physiological adaptations of the mother's body to cope with the physiological needs during lactation and pregnancy, including the high energy requirements of the developing fetus, followed by the production of milk to support the child after birth, have been attributed to PRL [13]. PRL plays a significant part in developing breast tumors and other hormonally sensitive malignancies such as pancreatic, lung, ovarian, and endometrial cancers [14]. PRL stimulates the growth of the mammary glands. According to a meta-analysis, elevated PRL levels were positively correlated with the development of breast cancer. The patient sub-group analyses also showed a positive correlation between PRL and invasive cancer of the breast and after-menopause status [15].

Relaxin

A class of peptide hormone/neuromodulators known as relaxins (RLNs) can control various physiological functions, including brain function and reproduction [16]. In pathological conditions like AF (atrial fibrillation) and cardiac failure, relaxin-2 has several beneficial cardiovascular benefits, although the exact processes by which it works are still poorly known [17]. Relaxin-2 plays a crucial function in many malignancies, as evidenced by the pro- and antitumor effects observed in various malignant tumors. However, this involvement is a double-edged sword. Given this dual function, the molecular mechanism behind relaxin-2's effect in cancer appears unclear, and more research to clarify its possible role as a cancer-preventative factor is of utmost relevance [18].

Hypothalamic and pituitary

Gonadotropin-Releasing Hormone (GnRH)

Human reproductive capability is initiated and maintained by the pulsatile production of the hypothalamic hormone GnRH. Congenital hypogonadotropic hypogonadism (CHH) is an uncommon condition that causes delayed puberty and infertility by impairing average episodic GnRH production [19]. The overall quality of the proof was deficient due to the considerable bias and indirect risk associated with nonrandomized studies. There was no appreciable increase in the incidence of breast cancer among women taking any type of stimulation of the ovaries medication for infertility when compared to unaffected controls from the entire population and the barren group. Furthermore, neither gonadotropins nor clomiphene citrate, either alone or in combination, significantly raised the possibility of breast cancer [20].

Luteinizing Hormone (LH)

Many females experience infertility due to reproductive dysfunction, which has drawn the interest of several researchers due to its complicated pathophysiology. Ovulation problems resulting from various pathogenic reasons are a defining feature of female infertility. Through decreased LH, p62 deficiency in the pituitary caused female infertility [21]. Eighty percent of newly discovered instances of breast cancer are hormone-dependent. These hormones have been shown to promote the growth and spread of tumors. In this context, preliminary data point to a potential function for LH in tumorigenesis. This hormone controls cell migration and invasion in BC cells that exhibit functional LH receptors (LHR) [22].

Follicle-Stimulating Hormone (FSH)

The pituitary gland produces FSH as part of a synchronized hypothalamic-pituitary-gonadal (HPG) axis event; it is crucial for fertility because it is essential for reproduction and the development of germ cells at various stages of reproductive development (fetal, adult, puberty, and neonatal life). The dissociable components comprise the heterodimeric glycoprotein hormone FSH [23]. To preserve fertility through mature oocyte cryostorage, breast cancer patients under the age of 40 who are candidates for treatment with alkylating agents may undergo controlled ovarian stimulation (COS) using recombinant human follicle-stimulating hormone, also known as rhFSH. According to observations, a brief exposure to rhFSH causes chemoresistance to doxorubicin (DOX) and cyproterone acetate (CPA) in human cancerous breast cells through activating hypoxia-inducible factor (HIF-1) [24].

Oxytocin (OT)

A pleiotropic peptide hormone called oxytocin extensively affects social behavior, development, reproduction, and overall health. Endogenous oxytocin promotes patterns of growth, resiliency, and healing, as does activation of the oxytocin receptor. An anti-inflammatory, antioxidant, and stress-coping chemical, oxytocin has protective benefits, particularly when faced with hardship or trauma [25]. Even while cancer mainly affects people in their later years, many patients-such as those with breast cancer-are identified with the disease before starting families or even before having children. In addition, cytotoxic chemotherapy could be necessary in addition to other treatments for cancer survivors. The goal of the current investigation was to determine if oxytocin (OT) can protect rats against methotrexate (MTX)-induced ovarian damage [26]. OT concentration and OT receptor (OXTR) expression changes have varying impacts on cells originating from breast cancer. This investigation was carried out to assess OT variation in breast carcinoma patients and OXTR expression alterations in breast cancer tissues. A reduced expression of OXTR in malignant tissues appears to be helpful in the evolution of breast cancer despite the elevated levels of OT concentration in breast cancer patients [27].

Ovary

Progesterone

A vital steroidogenic precursor for various non-gonadal and gonadal hormones, including aldosterone, estradiol, cortisol, and testosterone, progesterone is known to have a significant physiological role in both the menstrual cycle and pregnancy [28]. According to research, progesterone may have a role in the etiology of breast cancer, and there is interest in limiting progesterone activity to prevent or cure breast cancer [29].

Androstenedione

Dehydroepiandrosterone (DHEA), testosterone, dehydroepiandrosterone sulfate dihydrotestosterone, androstenedione are all naturally occurring androgens in females. Acne, hirsutism, and androgen-mediated cutaneous diseases (AMCDs) associated with female pattern hair loss (FPHL) are only a few of the frequent cutaneous problems that these androgens are crucial in the development [30]. The most prevalent steroid in circulation is DHEA sulfate (DS), found in breast fluid at a concentration of around 30 times higher than in serum. Organic anion transporting polypeptides (OATPS), which are specialled for cell type, cell location, and substrate but may have a broader specificity for d for housekeeping tasks, are required to transfer DS into cells. Androstenedione and testosterone, DS metabolites, are linked to breast cancer, but DHEA likely also has an independent impact [31]. It has been shown that the androgen receptor (AR) has a variety of functions in breast cancer. The conversion of only circulatory androgens into estrogens can be reduced by 99% with aromatase inhibitor (Al) therapy [32].

Estrogens

During pregnancy, the breast undergoes morphological and physiological changes that prepare it for lactation. The ductal system enlarges and branches into the fat tissue in response to the surge in estrogen throughout the first trimester. Another consequence of elevated estrogen levels is decreased adipose tissue and ductal proliferation and elongation [33]. The precise reasons for the higher likelihood of ER-negative breast cancer associated with estrogen usage are unknown. The fact that estrogen encourages the development of this particular kind of tumor suggests that, as opposed to the tumor cells themselves, estrogens have an impact on the tissues of the host that is carrying the cancer [34].

Inhibin

The transforming growth factor beta (TGFb) superfamily includes activins and inhibins, which include the isoforms of activin C, A, B, AB, and E, and inhibin A and B, respectively. They control various biological processes, such as cellular differentiation, proliferation, and invasiveness, to improve the synthesis and operation of several tissues in humans and organs [35]. According to the newly available information, inhibin subunit beta A (INHBA) is dysregulated and implicated in several malignancies. Studies have found that INHBA has been elevated in breast cancer tissues thanks to advances in sequencing technology. The biological roles of INHBA in breast cancer are yet unclear, though [36].

Reflects the predisposing and overall effect of placental, uterine, hypothalamic and pituitary, and ovarian hormones on breast cancer. We are dealing with the hormones somehow linked with the female reproductive system. HPL is supposed to mainly affect the breast. It involves the development of breasts and is believed to reduce the growth of breast cancer and is considered a protective hormone, thus its effect is protection from the development of breast cancer in the life of a female. The second hormone secreted from the placenta is human chorionic gonadotropin. This hormone does not have enough research data to prove its role in breast cancer but is supposed to increase the risk of development of breast cancer in females with higher levels of hGC and if the levels persist for a longer time in the body. The hormone prolactin is supposed to increase the risk of breast cancer development in a female's life. The increased levels of prolactin are supposed to be harmful to females and thought to increase the risk of development of breast cancer in the life of females. Thus disregulation of the hormone prolactin increases the risk of the development of breast cancer in the life of a female. Relaxin is the second hormone secreted by the uterus, which is supposed to have a preventive role against the development of breast cancer in the life of females. Still, the data is insufficient for it to be proved. However, this hormone relaxin is supposed to be considered a protective role for breast cancer. 

Hypothalamic and pituitary secret hormones gonadotropin-releasing hormone increases the risk of breast cancer development in a female's life, but it has insufficient data to prove its role; it is not a protective hormone for breast cancer. The following hormone, luteinizing hormone, secreted during the menstrual cycle, is supposed to help in the development of tumors in the breast. This tumorigenesis action of this LH hormone is considered to promote the growth of breast cancer in the life of a female. A follicle-stimulating hormone secreted during the menstrual cycle is supposed to prevent the action of certain drugs and causes chemoresistance; however, the data is insufficient to prove this. The fourth hormone, oxytocin, is believed to be protective against developing breast cancer. It causes alterations in the breast tissue and thus has a protective hormone effect in breast cancer. Ovaries secret progesterone during the life of a female. This hormone, if secreted in a limited amount, helps the breast protect against breast cancer. The critical part of this hormone is that its small amount helps cure breast cancer. Androstenedione's role is uncertain but is linked to breast cancer development. Estrogen, due to its encouraging role in the development of tumors, helps organs have the potential of turning malignant, and this increases the risk of breast cancer. Inhibin is a hormone of disregulated levels, which may increase the development of breast cancer in females; however, the role is uncertain and yet to be proved (Table 1).

**Table 1:** Effect of hormones on breast cancer

Sr. No.	Name of organ	Name of hormone secreted	Predisposing effect on breast cancer	The overall effect on breast cancer
1.	Placenta	Human placental lactogen (HPL)	Reduces the risk	Protective effect
		Human chorionic gonadotropin (hCG)	Conflicting data	Increases the chances
2.	Uterus	Prolactin	Increases the risk	Dysregulation increases the risk
		Relaxin	Prevents risk of cancer	Data insufficient
3.	Hypothalamic and pituitary	Gonadotropin-releasing hormone (GnRH)	Increases the risk	Data insufficient
		Luteinizing hormone (LH)	Tumorigenesis action	Promote growth
		Follicle-stimulating hormone (FSH)	Data insufficient	Causes chemoresistance
		Oxytocin	Protective effect	Alteration in tissue noted
4.	Ovary	Progesterone	Reduces the risk when in a limited amount	Limiting the amount helps in the cure.
		Androstenedione	Unclear	Linked
		Estrogens	Encourages tumour growth	Impacts tissue carrying tumour cells
		Inhibin	Dysregulated levels increase the risk	Unclear

## Conclusions

Cancer is the leading cause of death worldwide. Cancer is a malignant growth of cells that takes up the nutrition from the surrounding tissue and starves them to death. Cancer cells have escaped the normal cell cycle and do not undergo apoptosis. Cancer cells can spread all over the body from the primary site to the secondary site using various channels of our human body spreading through the blood, spreading through the body's lymphatic drainage, and many more. The factors that cause cancer are known as carcinogens. These carcinogens are the reason for the occurrence of cancer at the primary site in the human body. Carcinogens are major of three types chemical mutagens, physical mutagens, and biological mutagens. The physical mutagens include factors like radiation, and the natural mutagens include microorganisms like viruses. The chemical mutagens include carbon compounds, hormones, and other factors. This review article presents the hormonal effect on cancer predisposition and its overall development. We are dealing with the hormones somehow linked with the female reproductive system.
